# Cytochrome P450 3A4, 3A5, and 2C8 expression in breast, prostate, lung, endometrial, and ovarian tumors: relevance for resistance to taxanes

**DOI:** 10.1007/s00280-019-03905-3

**Published:** 2019-07-15

**Authors:** Maarten van Eijk, René J. Boosman, Alfred H. Schinkel, Alwin D. R. Huitema, Jos H. Beijnen

**Affiliations:** 1 Department of Pharmacy and Pharmacology, The Netherlands Cancer Institute, Plesmanlaan 121, 1066 CX Amsterdam, The Netherlands; 2 Division of Pharmacology, The Netherlands Cancer Institute, Plesmanlaan 121, 1066 CX Amsterdam, The Netherlands; 3 Department of Clinical Pharmacy, University Medical Center Utrecht, Utrecht University, Heidelberglaan 100, 3584CX Utrecht, The Netherlands; 4 Science Faculty, Utrecht Institute for Pharmaceutical Sciences (UIPS), Division of Pharmacoepidemiology and Clinical Pharmacology, Utrecht University, P.O. Box 80082, 3508 TB Utrecht, The Netherlands

**Keywords:** CYP, Paclitaxel, Docetaxel, Ritonavir, Intratumoral

## Abstract

Enzymes of the cytochrome P450 (CYP) subfamily 3A and 2C play a major role in the metabolism of taxane anticancer agents. While their function in hepatic metabolism of taxanes is well established, expression of these enzymes in solid tumors may play a role in the in situ metabolism of drugs as well, potentially affecting the intrinsic taxane susceptibility of these tumors. This article reviews the available literature on intratumoral expression of docetaxel- and paclitaxel-metabolizing enzymes in mammary, prostate, lung, endometrial, and ovarian tumors. Furthermore, the clinical implications of the intratumoral expression of these enzymes are reviewed and the potential of concomitant treatment with protease inhibitors (PIs) as a method to inhibit CYP3A4-mediated metabolism is discussed.

## Introduction

Breast, prostate, and lung cancer were among the top five most diagnosed cancers worldwide in 2018, while endometrial and ovarian cancer were the most common and deadly gynecologic malignancies in Europe [1]. Despite the emergence of new targeted therapies such as immunotherapy, hormonal therapies, tyrosine kinase, and poly (ADP-ribose) polymerase (PARP) inhibitors, the taxanes, docetaxel and paclitaxel, are still important drugs used in the treatment of these malignancies both as single agents and as part of combination regimens [2–4]. This applies especially in malignancies with fewer treatment options available, such as triple negative breast cancer (TNBC) and metastatic castration-resistant prostate cancer (mCRPC) [5–7]. Moreover, a significant increase in survival has been observed in patients with metastatic and non-metastatic hormone naïve prostate cancer treated with docetaxel in addition to androgen-deprivation therapy (ADT) in the CHAARTED and STAMPEDE trials [8, 9].

**Fig. 1:**
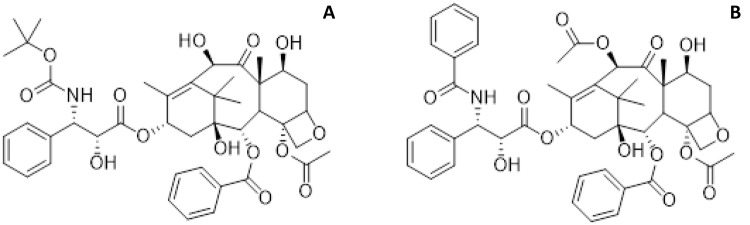
Chemical structures of the taxanes: **a** docetaxel and **b** paclitaxel

The taxanes (see Fig. 1) bind to the tubulin β subunit, where they stabilize the microtubules by precluding depolymerization. Thereby, cell arrest in the mitotic G2/M phase is induced, leading to cell death [10]. Although paclitaxel and docetaxel come from a similar class of chemotherapeutic agents, their pharmacological characteristics exhibit several differences. Compared to paclitaxel, docetaxel has a longer half-life, higher cytotoxicity, a lower schedule dependency, a different adverse effect profile, longer retention time, and higher in vivo accumulation in tumors [11, 12]. This has led to the more frequent use of docetaxel compared to paclitaxel [13]. Unfortunately, patients treated with docetaxel or paclitaxel will often develop resistance [14–18]. Interestingly, despite the similar structural characteristics of the two drugs, a lack of cross-resistance has been observed. For instance, docetaxel has shown activity in a number of paclitaxel-refractory solid tumors [19–21].

Various mechanisms by which resistance to taxane-based chemotherapy can arise have been proposed. These mechanisms can broadly be classified into the following: (1) pre-target events resulting in reduced intracellular drug concentrations, (2) alterations of the drug–target interaction, or (3) factors influencing the cellular response to damage of the cytoskeleton [22]. Pre-target events could, for example, involve upregulation of the ATP-binding cassette (ABC) drug efflux transporters such as P-glycoprotein (P-gp, ABCB1) or multidrug resistance protein (MRP1, ABCC1) and breast cancer resistance protein (BCRP, ABCG2) [23]. Furthermore, hepatic clearance through metabolizing enzymes from the Cytochrome P450 (CYP) superfamily can contribute to decreased plasma exposure. Both paclitaxel and docetaxel are mainly metabolized by CYP3A4. In addition, docetaxel is metabolized by CYP3A5 and paclitaxel by CYP2C8 [24–26].

CYP enzymes are also expressed in a variety of extrahepatic tissues. CYP3A, for example, is markedly expressed in the tissue of the digestive tract [27]. Similarly, it is known that various malignant tissues express CYP enzymes [28]. A variety of studies showed expression of CYP3A4 protein in breast, colorectal, esophageal tumors, and Ewing’s sarcoma [29–33]. This expression of CYP enzymes in tumors may limit the intracellular concentrations of docetaxel and paclitaxel, which may cause pre-target resistance. The metabolites of both taxanes show very little if any cytotoxic activity. It has previously been described that the major metabolite of paclitaxel in humans, 6α-hydroxypaclitaxel, does not induce growth inhibition in tumor cell lines [34]. Likewise, the metabolites of docetaxel show little-to-no antitumor activity [35]. The intratumoral expression of CYP enzymes could, therefore, limit efficacy or even contribute to the development of resistance to taxane therapy. This review will elaborate on the possible role of the CYP enzyme system in tumors of the breast, prostate, lung, ovaries, and endometrium in relation to the clinical pharmacology of docetaxel and paclitaxel. To this end, the expression of CYP enzymes in tumor tissue of different malignancies will be discussed, and possibilities for attenuation of CYP enzymes in tumors will be considered. To our knowledge, Oyama et al. were the first to review the intratumoral expression of CYP enzymes in 2004 [28]. In this review, we provide updated data on CYP3A4, CYP3A5, and CYP2C8 expression and review the impact of the intratumoral expression on taxane-based therapy.

## Drug-metabolizing CYP enzymes

CYP enzymes are found predominantly in the liver and intestines and serve as a clearance mechanism by catalyzing the degradation of exogenous and endogenous substances. Approximately 60 human CYP genes are known, consisting of 18 gene families and 43 subfamilies [36]. CYP enzymes have a broad spectrum of functionalities in relation to cancer. On one hand, these enzymes may protect against carcinogens and even play a role in the activation of anticancer agents. For example, cyclophosphamide, an alkylating prodrug used as immune suppressor and chemotherapeutic for a range of tumors, is metabolized by CYP2A6, CYP2B6, CYP3A4, CYP3A5, CYP2C9, CYP2C18, and CYP2C19 to its active metabolites 4-hydroxy-cyclophosphamide and aldophosphamide [37]. On the other hand, CYP enzymes may play a role in the activation of carcinogens and the metabolism of anticancer drugs. For example, CYP1B1 is overexpressed in many tumor types in comparison to normal tissue and is known for its ability to activate a variety of carcinogens such as polycyclic aromatic hydrocarbons (PAH), heterocyclic amines, aromatic amines, and nitropolycyclic hydrocarbons. Moreover, many anticancer agents are metabolized by the CYP enzyme system into their inactive form [38–40]. One of the most abundantly expressed CYP enzymes is CYP3A4 which is responsible for the degradation of more than 60% of all marketed drugs [41]. The regulation of its expression has been investigated in a number of studies but has not been fully elucidated. The human pregnane X receptor or steroid xenobiotic receptor (SXR) is the most frequently studied receptor for the control of the CYP3A4 expression [42–45]. Activation of this receptor occurs after binding with CYP inducers, such as rifampicin but also in response to endogenous steroids such as estradiol [46, 47]. Binding of the ligand to the receptor results in the dimerization of the SXR with the 9-*cis*-retinoid X receptor (RXRα). This heterodimer subsequently binds to its response element on the CYP genes resulting in the transcriptional activation of CYP enzymes [48]. In addition, SXR can enhance drug efflux through the induction of P-gp [49]. It is known that steroid-dependent neoplasms such as breast and endometrial cancer express higher levels of SXR in neoplastic tissues than normal tissues [50, 51]. Paclitaxel, through SXR, markedly induces expression of CYP3A4 and CYP2C8 and P-gp, thereby preventing its own uptake and increasing its own metabolism and excretion when given in a weekly dosing schedule [49, 52, 53]. Conversely, docetaxel does not appear to enhance the activity of CYP3A4, although it does activate the transcriptional activation of SXR and CYP3A4 mRNA in human hepatocytes. However, this effect is very weak in comparison to that of paclitaxel [52, 53]. If one of the above-mentioned mechanisms would similarly be present in tumor tissue, this could contribute to the development of resistance or unresponsiveness to chemotherapy in these cells.

## Expression of CYP3A4, CYP3A5, and CYP2C8 in tumors

The expression of drug-metabolizing CYP enzymes in human tumors and other extrahepatic tissues has been a subject of investigation for several years [54]. Due to the metabolism or activation of many anticancer drugs by CYP enzymes, it is of particular interest to investigate whether CYP enzymes are also expressed in tumor tissue [40, 55]. CYP expression in tumor and non-tumor tissues of the breast, colon, and lung has been thoroughly studied. However, tumor tissues such as the endometrium and prostate remain poorly investigated [28]. Methods often used to study the presence of CYP enzymes in tissues include immunohistochemistry (IHC) or western blot [56, 57]. In addition, the presence of mRNA can be measured using reverse transcription polymerase chain reaction (RT-PCR) or Northern Blotting [58, 59]. Studies investigating the presence of CYP3A4/5 and CYP2C in extrahepatic tissues and tumors are summarized in Table 1.

**Table 1:** Overview of literature studies reporting the expression of CYP3A and CYP2C protein or mRNA from patient samples

Study	References	CYP3A				CYP2C			
		Protein		mRNA		Protein		mRNA	
		Tumor (*n*)	Healthy (*n*)	Tumor (*n*)	Healthy (*n*)	Tumor (*n*)	Healthy (*n*)	Tumor (*n*)	Healthy (*n*)
Breast cancer									
Albin et al. (1993)	[68]	0% (12)	0% (12)	–	–	0% (12)	0% (12)	–	–
Hellmold et al. (1998)	[63]	–	0%(15)	75% (4)	73% (15)	–	0% (14)^c^	100% (4)	100% (15)
Murray et al. (1993)	[66]	22% (54)^a^	–	–	–	–	–	–	–
Huang et al. (1996)	[62]	–	–	15% (13)	73% (11)^a^ 82% (11)^b^	–	–	100% (13)	100% (11)
Yokose et al. (1999)	[67]	0% (6)	–	–	–	33% (6)	–	–	–
Iscan et al. (2001)	[64]	–	–	0% (8)^a^ 0% (4)^b^	0% (8)^a^ 0% (4)^b^	–	–	83% (6)	83% (6)
Miyoshi et al. (2002)	[101]	37% (38)^a,d^	–	–	–	–	–	–	–
El-Rayes et al. (2003)	[56]	+ (29)	+ (29)	–	–	–	–	–	–
Kapucuoglu et al. (2003)	[29]	100% (25)^a^	68% (25)^a^	–	–	–	–	–	–
Knüpfer et al. (2004)	[72]	–	–	–	–		–	100% (10)^c^	–
Schmidt et al. (2004)	[71]	100% (11)^a^ 0% (10)^b^	–	–	–		–	–	–
Miyoshi et al. (2005)	[102]	52% (31)^a^	–	–	–	–	–	–	–
Haas et al. (2006)	[70]	25% (393)^a,b^	–	–	–	–	–	–	–
Vaclavikova et al. (2007)	[65]	–	–	BLQ (40)^a^	BLQ (40)^a^	–	–		
Murray et al. (2010)	[32]	52% (170)^a,d,e^ 19% (170)^b,d,e^	–	–	–	30% (170)	–	–	–
Sakurai et al. (2011)	[103]	55% (42)^a^	–	–	–	–	–	–	–
Floriano-Sanchez et al. (2014)	[69]	+ (48)*^,a^	+ (48)^a^	–	–	–	–	–	–
Prostate cancer									
Murray et al. (1995)	[79]	61% (51)	–	–	–	25% (51)	–	–	–
Yokose et al. (1999)	[67]	0% (6)	–	–	–	83% (6)	–	–	–
Finnström et al. (2001)	[58]	–	–	11% (28)^a,f^ 86% (28)^b,f^	11% (28)^a,f^ 86% (28)^b,f^	–	–	–	–
Koch et al. (2002)	[74]	–	–	–	0% (47)^a^ + (47)^b^	–	–	–	–
Di Paolo et al. (2005)	[80]	–	58% (24)^a^ 54% (24)^b^	–	–	–	–	–	–
Moilanen et al. (2007)	[77]	–	100% (6)^b^	–	–	–	–	–	–
Bièche et al. (2007)	[54]	–	–	–	+ (32)^b^				
Leskelä et al. (2007)	[76]	0% (35)^b^	100% (10)^b^	0% (10)^b^	+ (10)^b^	–	–	–	–
Fujimura et al. (2009)	[81]	75% (107)^a^	93% (88)^a^	–	–	–	–	–	–
Mitsiades et al. (2012)	[75]	–	–	+ (146)^a,b^	+ (29)**^,a,b^	–	–	–	–
NSCLC									
Nakajima et al. (1994)	[85]	–	–	–	–	+ (27)	+ (11)	–	–
Kivistö et al. (1995)	[86]	25% (32)	34% (32)	–	–	–	–	–	–
Kivistö et al. (1996)	[87]	100% (8)	100% (8)	0% (8)^a^ 50% (8)^b^	0% (8)^a^ 100% (8)^b^	–	–	–	–
Anttila et al. (1997)	[88]	–	18,5% (27)^a^	–	13% (8)^a^ 100% (8)^b^	–	–	–	–
Macé et al. (1998)	[84]	–	–	–	0% (14)^a^ 93% (14)^b^	–	–	–	100% (14)
Yokose et al. (1999)	[67]	0% (18)				0% (18)			
Fujitaka et al. (2001)	[90]	–	–	+ (10)^a^	+ (10)^a^	–	–	+ (10)*	+ (10)
Bièche et al. (2007)	[54]	–	–	–	+ (6)^b^	–	–	–	–
Qixing et al. (2017)	[89]	74% (87)***^,a,d^ + (87)^b,d^	+ (87)^a^ + (87)***^,b^	–	–	–	–	–	–
Endometrial cancer									
Hukkanen et al. (1998)	[94]	–	–	–	57% (7)^a^ 43% (7)^b^	–	–	–	–
Yokose et al. (1999)	[67]	0% (12)				0% (12)			
Sarkar et al. (2003)	[95]	–	–	–	57% (23)^a^	–	–	–	–
Masuyama et al. (2003)	[50]	–	–	+ (20)^a^	–	–	–	–	–
Ovarian cancer									
Yokose et al. (1999)	[67]	0% (12)	–	–	–	0% (12)	–	–	–
Klose et al. (1999)	[91]	–	–	–	–	–	–	–	100% (1)
Downie et al. (2005)	[98]	91% (99)/ 80% (22)^a,g^ 66% (99)/ 55% (22)^b,g^	64% (13)^a^ 55% (13)^b^	–	–	17% (99)/ 10% (22)^g^	36% (13)	–	–
Bièche et al. (2007)	[54]	–	–	–	+ (15)^b^	–	–	–	+ (15)
DeLoia et al. (2008)	[97]	–	–	9% (47)^a^ 89% (47)^b^	–	–	–	69% (48)^c^	–

The percentages shown indicate the number of samples in which CYP enzymes could be detected, with in parentheses the total amount of samples/patients analyzed. (+) indicates CYP enzymes were expressed, but the exact number of positive samples was not described or presented as immunoreactivity score. (−) indicates not measured. *P* values indicate higher proportion, immunoreactivity score, or expression level compared to respective tumor/non-tumor sample, *P < 0.05, **P < 0.01, ***P < 0.001

*BLQ* below limit of quantification

^a^Only CYP3A4

^b^Only CYP3A5

^c^Only CYP2C8

^d^Percentage indicates a fraction of tumors with moderate/high expression

^e^Original data from publication received from the authors

^f^No distinction between tumor and non-tumor tissue

^g^Percentages for primary ovarian cancer and peritoneal metastases, respectively

### Breast cancer

CYP enzymes are responsible for the phase I metabolism of estrogen and, therefore, have a prominent role in the pathogenesis of breast cancer. In extrahepatic tissues, CYP1B1 is responsible for the conversion of 17β-estradiol (E_2_) into 4-hydroxyestradiol which may act as a carcinogen, while CYP1A1 and CYP3A4, on the other hand, metabolize E_2_ into its non-carcinogenic 2-hydroxy metabolite [60, 61]. This extrahepatic expression of enzymes may also have implications for treatment with taxanes. Studies using RT-PCR to detect CYP3A4 mRNA have produced variable results with some studies indeed finding relevant CYP3A4 expression [62, 63], and some others find no expression of CYP3A at all [64, 65]. Other experiments using IHC or western blot to detect CYP3A protein expression also produced contrasting results [63, 66–68]. When comparing expression levels in malignant versus healthy tissue, results are similarly ambiguous with some studies finding a lower CYP3A4 expression in malignant tissues compared to adjacent morphologically normal tissue [56], and other studies suggesting increased expression of CYP3A4 in tumors [29, 69]. In one of the larger trials investigating CYP expression in mammary tumors, Haas and colleagues analyzed tissue from 393 breast cancer patients using IHC. Their analysis showed expression in 25% of mammary tumor samples screened for CYP3A4/5. Moreover, this CYP3A4/5 expression showed a significant association with a positive nodal status in patients (*P* = 0.018) [70]. In 2010, Murray and colleagues [32] also found an association between CYP3A4 expression and survival. Although the difference was marginal, patients with tumors that showed a low/negative CYP3A4 immunoreactivity had a mean survival of 79 months (95% confidence interval (CI) 77, 81), while patients with tumors that showed moderate/strong CYP3A4 immunoreactivity had a mean survival period of 86 months (95% CI 79, 93) [32]. Some studies have investigated the mRNA and protein expression of enzymes of the CYP2C subfamily in breast cancer tumors with similar contradictory results [62, 63, 65, 67, 68, 71, 72]. Schmidt and colleagues, in addition to detecting CYP3A4 and CYP2C9 in breast cancer microsomes, also investigated the ability of these microsomes to metabolize ifosfamide. Using LC/MS, a minimal in vitro ifosfamide N-dechloroethylation (0.12 ± 0.07 pmol min^−1^ mg_protein_^−1^) could be detected in all four measured breast cancer microsomes. In comparison, previous investigation studies in liver samples from female patients had shown activities of 132 ± 57 pmol min^−1^ mg_protein_^−1^ for ifosfamide N-dechloroethylation [71, 73]. Although very minimal, this demonstrates that the mechanism of CYP3A4-mediated ifosfamide metabolism is present in breast cancer microsomes.

Despite the large variability in reported expression frequencies, some larger studies suggest that the CYP3A4 protein is present somewhere between 20 and 55% of breast cancer tissues. For CYP2C enzymes, there also appears to be some expression in mammary tissue, whereas, for CYP3A5, this evidence is very limited. Although, in the majority of studies, the functionality of the enzyme remains to be elucidated, the fundamental conditions for CYP mediated metabolism appear to be present in a subpopulation of breast cancers which may have implications for taxane chemotherapy.

### Prostate cancer

Interestingly, several studies which measured CYP3A mRNA in both normal prostate and cancerous tissue seem to suggest that CYP3A5 is the most abundant CYP in these tissues [54, 58, 74–77]. Even though about 80% of Caucasians are CYP3A5 deficient [78]. While studies investigating CYP3A protein expression in tumor samples have mainly found relatively high expression of both CYP3A4 and CYP3A5 both in tumor and non-tumor tissue [79–81]. Furthermore, enzymes of the CYP2C family were also detected in tumor samples in some studies [67, 79]. In 2009, Fujimura and colleagues detected CYP3A4 in healthy prostate and prostate cancer tissue and found that prostate cancer cells had a lower CYP3A4 immunoreactivity score (sum of the proportion of positively stained cells and staining intensity; 3.6 ± 2.6) compared to the benign epithelium (4.5 ± 2.1; *P* < 0.0001). Moreover, this lower immunoreactivity score showed a significant inverse correlation with a higher Gleason score and a poorer prognosis in patients [81]. This result was supported by the finding of a decreased expression of CYP3A4 and CYP3A5 in CRPC cells compared to benign prostate tissue [75]. Physiologically, this could be explained by a reduced conversion of androgens, such as testosterone into the inactive 6β-hydroxytestosterone (6β-OH-T) metabolite, leading to increased androgen-dependent proliferation. A hypothesis is supported by the association between CYP3A4 and CYP3A5 polymorphisms and haplotypes and prostate cancer risk and aggressiveness [82, 83]. In conclusion, heterogeneous CYP3A4, CYP3A5, and CYP2C8 expression in neoplasms of the prostate is observed, possibly contributing to variable treatment response to taxanes, even though the expression may be decreased in malignant tissue in comparison to healthy tissue.

### Non-small cell lung cancer (NSCLC)

RT-PCR analyses have shown that CYP3A4 and CYP3A5 are present in healthy and malignant lung tissue [54, 84, 85]. Yet, results of IHC analyses are less clear, although the CYP3A4 protein is expressed in about 20% of the observed tissue samples [86–88]. A more recent study showed that CYP3A4 expression was significantly higher in tumor tissue when compared with normal lung tissue [89]. These results, obtained from an online data set on CYP3A4 and CYP3A5 expression, did not contain data whether the patients received prior chemotherapy or not. Therefore, the study also featured an IHC analysis of 92 patients, who were included prior to any chemotherapy treatment. In this subset, a significant higher CYP3A4 expression was observed in comparison with the adjacent healthy tissue. In addition, CYP3A4 expression was significantly correlated with advanced TNM stages (*P* = 0.013) and poor histological differentiation (*P* = 0.017), while CYP3A5 was only significantly associated with histological differentiation. Moreover, an association between high-CYP3A4 or low-CYP3A5 expression and poor survival could be observed [89]. CYP2C gene-expression levels were found to be significantly increased in lung cancer tissue compared to healthy lung tissue [90]. In the study by Klose et al, CYP2C8 mRNA expression was found to be highly variable, although some older studies were able to detect CYP2C8 protein or mRNA [84, 85, 91]. In conclusion, taxane-metabolizing enzymes appear to be present in both healthy and malignant lung tissue, and upregulation of these enzymes may be observed in malignant tissue.

### Endometrial cancer

Estrogen itself is an important contributor to the growth and development of endometrial tumors. Contrary to the effects of progesterone, estrogen stimulates the endometrium to proliferate. A misbalance in favor of estrogen may, therefore, contribute to the early stages of endometrial cancer formation [92]. The extrahepatic metabolism of estrogen by CYP1B1, 1A1, and CYP3A4 is described above. As in breast cancer, these enzymes may also be present in endometrial tumors and play a role in local estrogen metabolism. The presence of CYP3A4 and CYP3A5 enzymes in endometrial cells seems variable with some studies finding no CYP3A4 and CYP3A5 mRNA, but high expression of CYP3A7 mRNA in the endometrium and placenta [93]. In contrast to this finding, other studies found expression of CYP2C, CYP3A4, CYP3A5, and CYP3A7 mRNA among other CYP enzymes in normal endometrium tissue [94, 95]. Another study found that CYP3A4 and CYP3A7 mRNA expression was low in normal endometrium, but was significantly upregulated in endometrial cancer tissues [50]. Together, this suggests some expression on an mRNA level of taxane-metabolizing enzymes of the CYP3A subfamily in healthy endometrium and endometrial cancer, although the small body of evidence does not allow for any strong conclusions.

### Ovarian cancer

As in breast and endometrial cancer, it is thought that estrogen plays a similar role in tumor initiation and promotion in ovarian cancer [96]. CYP enzymes may, therefore, also play a similar role in ovarian tumors. The presence of CYP2C8 and CYP3A5 mRNA has been reported in ovarian tissue [54, 91]. However, mRNA in both studies was collected from the whole gland tissue, whereas ovarian tumors are mainly of epithelial origin [97]. Downie et al. found that CYP3A5, among other CYP enzymes, had a significantly greater intensity of IHC staining (*P* < 0.001) in primary ovarian cancer tissue compared with normal ovary [98]. A later study investigated the presence of taxane-metabolizing enzymes in ovarian cancer and found that CYP3A4 is expressed at very low levels in ovarian cancer, while CYP3A5 and CYP2C8 were expressed in the majority of ovarian tumors, regardless of histologic type, stage, or grade [97]. As in endometrium, evidence regarding the expression of taxane-metabolizing CYP enzymes in the ovaries and in ovarian cancer is limited. Although the studies available seem to suggest a relatively high expression compared to other tissues, especially for CYP3A5.

## Taxanes and CYP3A expression in tumor cells

The hepatic induction of CYP3A enzymes by paclitaxel and to a lesser degree by docetaxel prompts questions whether a similar mechanism could have an effect on the expression of CYP3A enzymes in tumors [52]. This mechanism may be of clinical relevance during the application of taxane chemotherapy as it may impact treatment outcome [49, 52, 53]. In vitro studies have shown that human prostate cancer (DU-145) and breast cancer (MCF-7) cells lines indeed express a higher amount of CYP3A4 protein in response to treatment with docetaxel [99, 100]. In addition, Ikezoe and colleagues found a 2.0-fold increase in CYP3A4 expression in DU-145 xenografts in BNX nude mice after treatment with docetaxel [99]. Fujitaka and colleagues observed an increase in CYP3A4 mRNA expression in peripheral mononuclear cells from patients with previously untreated lung cancer after treatment with docetaxel. For CYP2C8, no such increase could be observed [90]. Similarly, DeLoia et al. investigated gene expression of CYP2C8, CYP3A4, CYP3A5, and ABCB1 in epithelial ovarian tumors, and exposed these tumor cells to docetaxel and paclitaxel ex vivo. There was no apparent correlation between any single gene expressed and taxane disposition, although a strong correlation between the ratio of CYP3A5:ABCB1 and the clearance of docetaxel was observed [97].

The presence of CYPs in tumors can similarly be linked to clinical outcomes of docetaxel treatment. Miyoshi et al. found that CYP3A4 expression in tumors, measured by mRNA and IHC, correlates with clinical outcomes in breast cancer patients treated with docetaxel. Patients with low CYP3A4 mRNA levels (*n* = 14) exhibited a significantly higher response rate to docetaxel treatment than those with high CYP3A4 mRNA levels (*n* = 9, 71% vs. 11%, *P* < 0.01) [101]. In addition, patients with CYP3A4-negative tumors (*n* = 15), determined by IHC, showed a significantly higher response rate to docetaxel treatment than those with CYP3A4-positive tumors (*n* = 16, 67% vs. 19%, *P* < 0.01) [102]. Later, breast cancer tissue obtained from a larger trial in 42 patients who underwent docetaxel treatment as adjuvant chemotherapy after surgery was analyzed for CYP3A4 expression using IHC. The 19 patients with CYP3A4-negative tumors showed a significantly higher response rate to docetaxel treatment than the 23 patients with CYP3A4-positive tumors (63.2% vs. 26.1%, *P* < 0.01). Moreover, a higher clinical benefit rate was observed in CYP3A4-negative tumors (73.7% vs. 26.1%, *P* < 0.01) as well as a longer time to progression (8.9 ± 5.8 months vs. 5.2 ± 4.4 months, *P* < 0.05). These results suggest that assessing CYP3A4 expression in breast cancer may be a relevant tool to predict the response of the tumor to docetaxel treatment [103]. In 16 patients with NSCLC receiving docetaxel or docetaxel and carboplatin for advanced disease, CYP3A4 gene expression in peripheral mononuclear cells was analyzed. After 24 h, the CYP3A4 expression was significantly increased when compared to baseline. Treatment with carboplatin monotherapy did not cause any statistically significant difference in CYP3A4 expression. In the same study, 20 autopsy samples (10 NSCLC + 10 control) from chemotherapy-naïve patients were analyzed on the levels of CYP3A4 gene expression. Although the variability in gene expression was high, there was no significant difference between healthy and cancerous tissue. In the case of CYP2C8, however, increased expression in tumor tissue could be observed [90].

Despite the small number of patients included in these studies, the evidence presented seems to indicate that an increase in intratumoral CYP3A4 expression can be observed after treatment with taxanes. Increased CYP3A4 expression could be inversely correlated to clinical response rates to these drugs. Together, this suggests that these CYP enzymes are part of a resistance mechanism in which the in situ metabolism of docetaxel is accelerated, thereby diminishing response to docetaxel-containing chemotherapy.

## Concomitant treatment with taxanes and HIV-protease inhibitors

As a notorious group of CYP3A4 inhibitors, it is of great interest to know whether or not HIV-protease inhibitors (PIs) will have an effect on intratumoral CYP3A4 functionality. Ritonavir, developed as an HIV PI, is one of the most potent inhibitors of CYP3A4 known, although the precise mechanism of inhibition has yet to be clarified [104, 105]. Ritonavir is used in HIV therapy to boost the concentration of other drugs with a known CYP3A4-dependent metabolism [106]. Its effect on CYP3A4 is irreversible, and consequently, the reversal of inhibition is dependent on the degradation half-life of CYP3A4, which is thought to be about 29 h. After cessation of ritonavir treatment, CYP3A4 expression returns to baseline after approximately 6 days [107, 108]. Docetaxel is strongly metabolized by CYP3A4, and hence, it is hypothesized that concomitant treatment of docetaxel and ritonavir will increase the antitumor activity of docetaxel [99]. Pharmacokinetic parameters of docetaxel such as clearance and half-life are decreased and increased, respectively, when co-administered with ritonavir [109–111]. Several in vivo studies have shown the effect on the tumor response after co-administration with ritonavir [99, 112]. In one study with an immunocompetent, orthotopic Cyp3a^−/−^ mouse model, the effect of intravenous docetaxel and oral ritonavir on Cyp3a expressing K14cre; Brca1^F/F^; p53^F/F^ mammary tumors was studied [112]. The co-treatment led to a decrease in tumor volume greater than docetaxel treatment alone (70% vs. 30% shrinkage of the initial tumor volume after 3 weeks of treatment). In addition, the median time in which the tumor reached the critical tumor size (approximately 1500 mm^3^) was significantly increased when docetaxel and ritonavir were given together (65.6 ± 8.6 days vs. 53.6 ± 1.5 days for docetaxel monotherapy). As expected, the plasma concentration of docetaxel did not show significant differences in the ritonavir co-administered group. However, the intratumoral docetaxel concentration was significantly higher after 9 days of treatment with docetaxel and ritonavir in comparison with docetaxel monotherapy. Furthermore, the docetaxel metabolite concentrations were lower in the combination treatment group compared to the group treated with single-agent docetaxel, suggesting that ritonavir specifically inhibited the intratumoral metabolism of docetaxel [112].

PIs do not only interfere with the metabolism of taxanes by direct inhibition of CYP3A4, but could also amplify their antitumor effects via additional mechanisms. Table 2 summarizes the proposed synergistic effect of the PIs to docetaxel treatment. Using western blot analysis, it was shown that ritonavir could potentiate the effect of docetaxel on the activation of caspase-3 and the cleavage of PARP (which is cleaved as a late event during apoptosis). Ritonavir can inhibit the docetaxel-induced increase in CYP3A4 mRNA completely in the mouse androgen-dependent prostate cancer cells. In vivo docetaxel markedly decreased the growth rate and size of DU145 tumors in male BNX mice. Ritonavir alone showed no statistical significance either in growth or in weight of the tumors. Interestingly, the combination of the taxane and the PI showed an additional statistically significant decrease in both growth and tumor weights in comparison with the monotherapy of docetaxel. Histologically, a site composed of necrotic and fibrotic tissue was observed, but no cells of cancerous origin were detected. Moreover, the organs of the mice were not affected. In addition, ritonavir has shown to block the DNA-binding activity of NFκB in the DU145 cells and in vivo [99].

**Table 2:** Proposed synergistic effects of protease inhibitors (PI) to docetaxel treatment examined in in vitro studies

Cell line	PI	Proposed effect	References
NCI-H460 NCI-H520	Nelfinavir	Nelfinavir-induced inhibition of Akt signaling leading to more sensitivity to docetaxel	[114]
DU145 cell line	Ritonavir	Increased effect of docetaxel on activation of caspase-3 and cleavage of PARP	[99]
DU145 cell line	Ritonavir	Reduced DNA-binding activity of NFκB, surpassing one resistance mechanism of docetaxel	[99]
DU145 cell line	Ritonavir	Blocked the docetaxel-induced increase in CYP3A4 mRNA, decreasing the metabolism of docetaxel	[99]

In various types of cancer including prostate cancer, hyperactivity of the NFκB pathway has been observed [113]. This hyperactivity often results in the development of resistance to several anticancer drugs such as paclitaxel and docetaxel [99, 112]. By decreasing the DNA-binding activity of NFκB, it is possible that ritonavir surpasses this resistance mechanism of docetaxel and will, therefore, increase the effectiveness of the docetaxel treatment. In the lung cancer cell line NCI-H460, a decrease in growth of 39 and 21% was observed when treated with single-agent nelfinavir and docetaxel, respectively. However, when the cell lines were incubated with nelfinavir prior to docetaxel, a growth inhibition of 51% was observed. Similar effects were observed in the NCI-H520 cell line, suggesting that the nelfinavir-induced inhibition of the Akt signaling results in more sensitivity to docetaxel treatment [114]. The activation of Akt pathway is described in the literature as being responsible for the development of resistance, and as a result, tumors are often overexpressing Akt in these cell populations [115]. Moreover, docetaxel resistance is tackled in some studies by inhibiting the Akt pathway and therefore resensitizing the tumor cells for docetaxel treatment [116, 117]. Whether ritonavir acts the same way as nelfinavir on NSCLC cells is not studied in the earlier mentioned article. Yet, there are reports that ritonavir can block the Akt signaling in ovarian cancer and breast cancer and, thus, can re-sensitize the resistant tumor cells for docetaxel treatment [118, 119].

In summary, the co-treatment of docetaxel with ritonavir enhances the cytotoxic activity of docetaxel in the tumor and ritonavir could, therefore, potentiate the effect of docetaxel as a chemotherapeutic agent. Currently, the first Phase I and II trials using an oral formulation of docetaxel called ModraDoc006 co-administered with ritonavir are underway in mCRPC (NCT03136640) and metastatic breast cancer (NCT03890744). Earlier trials with this formulation in patients with various solid tumors have shown promising antitumor activity and highlight the potential of this innovative way of attenuating CYP activity to boost oral docetaxel bioavailability and to possibly improve clinical efficacy [120–122].

## Discussion

Although evidence remains slightly contradictory and many studies are limited by low sample sizes, there appears to be relevant expression of taxane-metabolizing enzymes CYP3A4, CYP3A5 and CYP2C8, among other CYP enzymes, in some malignant and non-malignant tissues of the breast, prostate, lung, endometrium, and ovaries [29, 32, 56, 58, 62, 63, 66, 67, 69–72, 76, 77, 79–81, 84, 86–89, 94, 95, 97, 98, 101–103]. Individual studies in NSCLC, and breast and ovarian cancer show increased expression in malignant versus non-malignant tissue [69, 89, 98], whereas, in prostate cancer, this ratio may be decreased [76, 81]. What is important to note, however, is that mRNA expression does not necessarily correlate with protein expression, and that protein detection by IHC discloses no information on the functional status of an enzyme. CYP3A4 appears to be upregulated as a response to docetaxel in a preclinical setting [90, 99]. Moreover, CYP3A4 expression can function as a predictor of the efficacy of docetaxel chemotherapy [101, 102].

Other than the synergistic mechanisms described above, PIs may contribute to inhibition of tumor proliferation through intrinsic antitumor effects [109]. Several mechanisms have been described by which these inhibitors are capable of reducing cancer growth [123]. For example, in breast cancer cells, ritonavir has been shown to inhibit heat shock protein 90 (Hsp90) and Akt, thereby inhibiting the growth of these cells [118]. In the androgen-independent prostate cancer cell lines, DU-145 and PC3, the HIV PIs saquinavir, ritonavir, and indinavir were effective in inhibiting the proliferation of these cells in a dose-dependent manner. Ritonavir was most prone to inhibit the proliferation, showing a 50% decrease in the growth of DU145 (half maximum inhibitory concentration (IC50) of 3 × 10^−6^ mol/L) and PC-3 cells (IC50 of 8 × 10^−6^ mol/L). However, the studied concentrations are approximately 1000-fold higher than the concentrations observed after standard dosage regimens of ritonavir in humans, raising the question whether the inhibition of proliferation can be observed in clinical practice [99, 124].

Although this review focusses specifically on PIs as a boosting strategy, the effect of other CYP3A4 inhibitors on the intratumoral concentrations of docetaxel may be an interesting topic for further study. One such example is cobicistat, specifically developed as a boosting agent, and an equally potent but more specific inhibitor of CYP3A4 than ritonavir, without inducing properties. Consequently, it might have fewer unwanted drug–drug interactions [125].

Considering the currently available information, in addition to hepatic CYP enzymes, intratumoral enzymes may play a role in the in situ metabolism of taxane chemotherapy and could, therefore, present an important factor influencing the outcomes of treatment. In the future, more systematic analysis of CYP expression in tumors may be a tool by which treatment response may be predicted or function as a criterion by which patients may be selected for treatment. In conclusion, the attenuation of CYP enzymes in tumors appears to be an interesting area of research through which the clinical benefit of anticancer agents may be potentiated.
